# Effectiveness of Data-Driven Quality Improvement on Hospitalizations and Health Outcomes for People With Coronary Heart Disease in Primary Care (QUEL): A Cluster Randomized Controlled Trial With 24-Month Follow-Up

**DOI:** 10.1161/CIRCOUTCOMES.125.012904

**Published:** 2026-04-15

**Authors:** Julie Redfern, Nashid Hafiz, Qiang Tu, Andrew Knight, Charlotte Hespe, Clara K. Chow, Tom Briffa, Robyn Gallagher, Christopher M. Reid, David L. Hare, Deborah Manandi, Nicholas Zwar, Mark Woodward, Stephen Jan, Emily R. Atkins, Tracey-Lea Laba, Elizabeth Halcomb, Laurent Billot, Tim Usherwood, Karice Hyun

**Affiliations:** Institute for Evidence-Based Healthcare (J.R.), Bond University, Gold Coast, Australia.; Faculty of Medicine and Health (J.R.), Bond University, Gold Coast, Australia.; Faculty of Health Sciences & Medicine (N.Z.), Bond University, Gold Coast, Australia.; Faculty of Medicine and Health (J.R., N.H., Q.T., C.K.C., R.G., D.M., T.U., K.H.), The University of Sydney, Australia.; Susan Wakil School of Nursing and Midwifery (J.R., R.G.), The University of Sydney, Australia.; School of Health Sciences, Faculty of Medicine and Health, The University of Sydney, Australia (A.K.).; University of New South Wales, Sydney, Australia (A.K.).; The University of Notre Dame, School of Medicine, Sydney, Australia (C.H.).; Westmead Applied Research Centre, University of Sydney, Australia (C.K.C., T.U.).; Western Sydney Local Health District, Australia (C.K.C.).; School of Population and Global Health, The University of Western Australia, Perth, Australia (T.B.).; School of Population Health, Curtin University, Perth, Australia (C.M.R.).; School of Public Health and Preventive Medicine, Monash University, Melbourne, Australia (C.M.R., D.L.H.).; Faculty of Medicine, Dentistry and Health Sciences, University of Melbourne, Australia (D.L.H.).; Department of Cardiology, Austin Health, Heidelberg, Victoria, Australia (D.L.H.).; The George Institute for Global Health, University of New South Wales, Sydney, Australia (M.W., S.J., E.R.A., L.B., T.U.).; Centre for Health Economics Research and Evaluation, University of Technology, Sydney, Australia (M.W., T.-L.L.).; School of Nursing, University of Wollongong, Australia (E.H.).; Department of Cardiology, Concord Hospital, Sydney, Australia (K.H.).

**Keywords:** adult, cardiac rehabilitation, coronary disease, primary health care, quality improvement, secondary prevention, telemedicine

## Abstract

**BACKGROUND:**

This trial aimed to test the effectiveness of a data-driven quality improvement program in primary care on cardiovascular hospitalizations, major adverse cardiovascular events (MACE), risk factor profiles, and medication prescriptions at 24 months in people with coronary heart disease (CHD) compared with standard care.

**METHODS:**

A single-blind, cluster randomized controlled trial recruiting Australian primary care practices (2019–2022) was conducted. Practices using compliant data extraction software and having ≥200 adult patients annually with CHD were the units of randomization, and adults with CHD (who visited their general practitioner in the past 12 months) were the units of analysis. Practices were randomized to intervention (12-month data-driven quality improvement including benchmarking, monthly reporting, and improvement planning) or control (standard care). The primary outcome was the proportion of participants who had unplanned cardiovascular disease hospitalizations at 24 months. Secondary outcomes were MACE, medication prescriptions, risk factor targets, and management planning. Data were extracted from electronic records linked to administrative data.

**RESULTS:**

A total of 51 primary care practices participated, resulting in a patient cohort of 7864. The mean age of the patient cohort was 71.9 (±11.8) years, 68% were men, and 24% had a prior myocardial infarction. At 24 months, there was no significant difference between the groups for unplanned cardiovascular disease hospitalizations (relative risk, 0.91 [95% CI, 0.75–1.10]; MACE, 0.81 [95% CI, 0.61–1.07]; prescription of antiplatelet, 0.94 [95% CI, 0.79–1.13]), statin, 1.03 [95% CI, 0.97–1.09], angiotensin-converting enzyme or angiotensin receptor blocker, 1.00 [95% CI, 0.93–1.07]; risk factor targets for low-density lipoprotein cholesterol, 0.99 [95% CI, 0.86–1.13], systolic blood pressure, 0.97 [95% CI, 0.87–1.09], or smoking, 0.96 [95% CI, 0.57–1.59]; or management planning, 1.02 [95% CI, 0.64–1.63]).

**CONCLUSIONS:**

A primary care, data-driven quality improvement program did not improve unplanned hospitalizations, MACE, medication prescriptions, achievement of risk factor targets, or management planning for people with CHD. Robust evidence for the use of a data-driven, collaborative approach to improving care for people with CHD in primary care remains elusive.

**REGISTRATION:**

URL: https://www.anzctr.org.au; Unique identifier: ACTRN12619001790134.

**What is Known**

- The potential of collaborative quality improvement has been evaluated in a variety of health care settings including acute care hospitals and primary care.
- Evidence for effectiveness has predominantly focused on surrogate end points, with very few having robust study designs.

**What the Study Adds**

- As a cluster randomized controlled trial, QUEL (Quality Improvement in Primary Care to Prevent Hospitalizations and Improve Effectiveness and Efficiency of Care for People Living With CHD) tested the effectiveness of data-driven quality improvement on secondary prevention of coronary heart disease in primary care with 24-month follow-up.
- No significant improvement was found in unplanned cardiovascular disease hospitalizations, major adverse cardiovascular events, cardiovascular disease risk factors, or medication prescriptions.
- The use of a collaborative approach to improving care for people with coronary heart disease and using routinely collected primary care data is complex to deliver, and evidence remains elusive.

**See Editorial by McClintick et al**

Cardiovascular disease (CVD), primarily coronary heart disease (CHD), remains the leading cause of death and hospital admissions globally.^1^ With medical advances and improved treatments, CHD death rates are reducing.^2^ Concomitantly, with an aging population, the number of people worldwide living with CHD in need of secondary prevention is increasing.^3^ However, despite widespread recommendations in international guidelines,^4–6^ adherence, access, and sustainability of secondary prevention strategies are suboptimal.^7^ Use of evidence-based secondary prevention medications and lifestyle changes declines in the initial 6 months^8^ after an acute nonfatal event, and access to cardiac rehabilitation remains poor.^9^ Taken together, there is an escalating need for improved efficiency of and access to effective secondary prevention strategies to reduce hospitalizations and health care costs.^3,7,9^

The World Heart Federation Roadmap for Secondary Prevention of CVD identifies primary care as offering the most inclusive, equitable, cost-effective, and efficient environment for optimizing physical, mental, and social well-being.^7^ Collaborative quality improvement for health care emerged some 30 years ago and was based on the concept of the Breakthrough Series developed by The Institute for Healthcare Improvement.^10^ The approach is designed to help organizations improve processes and care through collaborative efforts founded on shared learning from each other and recognized experts to make health improvements.^10^ Breakthrough Series Collaboratives are designed as a short-term (6–15 months) learning system that brings together clinical teams to seek improvement in a focused topic area.^10^ Within a collaborative, teams apply quality improvement methods, attend workshops, share learnings, undertake rapid cycle change actions, and are supported by expert facilitators.^10^

Collaborative improvement methodology has been evaluated in a variety of health care areas,^11^ including asthma,^12^ chronic heart failure,^13^ and compliance with health care standards.^14^ While such programs have been evaluated, evidence for their effectiveness has predominantly focused on surrogate end points, with further robust evidence needed to assess the efficacy of this method for improving care.^11–13^ Concomitantly, advances in the routine collection and integration of electronic records have enabled more advanced and efficient data availability.^15^ However, current research has not rigorously evaluated the effectiveness of data-driven collaborative quality improvement in primary care in the context of cardiovascular health. The QUEL trial (Quality Improvement in Primary Care to Prevent Hospitalizations and Improve Effectiveness and Efficiency of Care for People Living With CHD) aimed to test whether a 12-month data-driven quality improvement program, delivered in primary care, would reduce the rate of unplanned cardiovascular hospitalizations and improve cardiovascular risk profiles at 24 months among people with CHD. The trial also set out to test the effect on major adverse cardiovascular events (MACE), risk factor profiles, and medication prescriptions at 24 months.

## Methods

### Data Sharing Statement

Due to constraints related to ethical approval for the QUEL trial, participant-level data cannot be shared outside the Secure Unified Research Environment.

### Study Design and Participants

The QUEL trial was an investigator-initiated, pragmatic, multicentre, prospective, single-blind cluster randomized controlled trial with 12- and 24-month follow-up. The protocol is published elsewhere.^16^ The trial (ACTRN12619001790134) complied with the Consolidated Standards of Reporting Trials (CONSORT) extension for cluster randomized controlled trials^17^ and the intervention is reported in line with the Template for Intervention Description and Replication.^18^ The trial was conducted at 51 primary care practices across 4 of the most populous states of Australia (New South Wales, Victoria, Queensland, South Australia) between 2019 and 2022. Participating practices were randomized (1:1) to the intervention (12-month data-driven quality improvement program that included benchmarking, monthly reporting, and improvement planning) or control (standard care) regimens. The QUEL trial complied with the Declaration of Helsinki and was approved by the New South Wales Population and Health Services Research Ethics Committee (HREC/18/CIPHS/44) and appropriate regulatory agencies for linkage of administrative data.

Primary care practices were eligible if they (1) managed ≥200 patients per year with CHD to ensure that each practice had a sufficiently large eligible population to feasibly recruit the sample size needed per cluster, (2) used practice software that was compliant with the data extraction software (PEN Computer Systems) used for collection of clinical data, and (3) were not participating in a formal quality improvement collaborative program. The patient cohort comprised all eligible patients presenting to participating practices who were (1) >18 years of age, (2) had a documented diagnosis of CHD in the practice records, and (3) had visited their general practitioner at least once in the previous 12 months.

### Randomization and Masking

Practices were randomized 1:1 using a computer-generated sequence generated with SAS 9.4 (Proc Surveyselect). Randomization was stratified according to 2 characteristics—rural versus urban location and size of the practice (≤2 versus >2 general practitioners in a practice). The statistician performing randomization and analysis was blinded to practice names and details and was only exposed to the practice characteristics to enable stratification. All study data were obtained via linkage between administrative data sets, and no people involved in data linkage or analysis were aware of the group allocation. Practices and the research team members coordinating the intervention (NH, QT, AK, CH) could not be masked to allocation status but did not have access to the data or participate in the analysis.

### Procedure

Eligible practices that agreed to participate entered into a formal agreement, and baseline individual-level clinical data were extracted from practice software before randomization. Individual-level clinical data were also extracted for all included patients from the primary care electronic medical record at 12 and 24 months. Data were securely linked with Australian government administrative data, including the Pharmaceutical Benefits Scheme for subsidized medication prescriptions, Medical Benefits Scheme (MBS) for health care utilization, National Death Index for deaths, and relevant state government administrative data for hospitalizations. All data were then combined and analyzed in the Secure Unified Research Environment. Following randomization, practices allocated to the control arm continued with their usual care for all patients attending their practice.

#### Intervention

Practices allocated to the intervention arm participated in a 12-month data-driven quality improvement collaborative based on Breakthrough Series Methodology (Table 1).^10^ The intervention was implemented at the practice level and aimed to uplift care and processes for all patients visiting the practice. The intervention aimed to address predetermined key performance indicators (KPIs) relevant to secondary prevention of CHD.^16^ These consisted of measures related to LDL (low-density lipoprotein) cholesterol, blood pressure (BP), smoking status, medication prescriptions (antiplatelet, statin, angiotensin-converting enzyme inhibitor or angiotensin II receptor blockers), and documentation of a CHD General Practitioner Management Plan.^16^ These KPIs are included as secondary outcomes of the trial; however, there were also KPIs for documented records of LDL, systolic BP, and smoking status, but these were not included as study outcomes because they were included to facilitate practice recording of data. The intervention included participation in learning workshops by practice staff, preparation and submission of rapid improvement (plan-do-study-act [PDSA]) cycles using practice data between workshops, and support from local network members to provide technical assistance and reminders (Table 1).

**Table 1.**
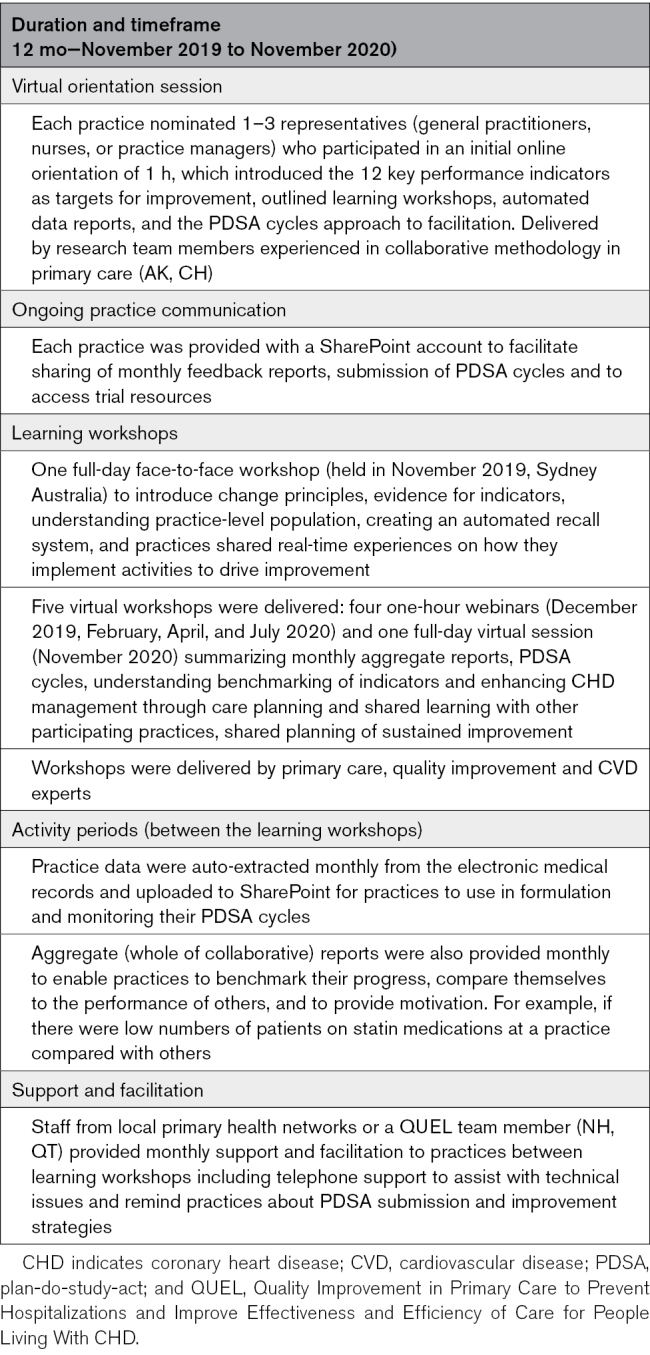
Overview of QUEL Data-Driven Quality Improvement Intervention

Six learning workshops were delivered during the intervention period (November 2019–November 2020). The first and sixth workshops were initially planned as full-day, face-to-face sessions; however, due to the rapid onset of the COVID-19 pandemic, only the first workshop was delivered in-person. Each participating practice nominated 1 to 3 representatives (eg, general practitioners, practice nurses, or practice managers) to attend the workshops and lead quality improvement activities within their practice. These representatives were responsible for disseminating information and resources from the workshops to their wider practice team.

Monthly feedback reports were generated for each practice based on their electronic health record data aligned with the 12 KPIs and shared via a secure SharePoint portal. Between learning workshops, practices were encouraged to independently review these reports and use the findings to develop and implement their own rapid improvement cycles (PDSA) tailored to their local context, patient population, and improvement priorities. Although the extent of individual report review was not formally tracked, aggregate results and examples of practice-level improvement were discussed during subsequent learning workshops to facilitate shared learning, benchmarking, and sustained engagement across practices. Ongoing support was provided monthly by staff from participating Primary Health Networks and the QUEL study team, who assisted practices with data-related queries, encouraged submission of PDSA cycles, and guided the implementation of improvement strategies.

### Outcomes

The primary outcome was the proportion of patients with at least 1 unplanned CVD hospitalization within 24 months from baseline data collection. For this trial, CVD was defined as any condition involving the heart, brain, or peripheral blood vessels and included CHD (such as angina and myocardial infarction), cerebrovascular disease (such as stroke), peripheral arterial disease, heart failure, and atrial fibrillation.^19^ (Primary outcome data were collected via probability-matched and privacy-preserved linkage of the practice cohorts with individual-level state/territory government administrative data for hospital admissions (Table S1).

Secondary outcomes were:

1. Proportion of patients with MACE that included CHD (unstable angina or myocardial infarction), stroke, or CVD death collected via probability-matched and privacy-preserved linkage of the practice cohort with individual-level state government hospital readmissions and federal government death index administrative data at 24 months (Table S1 for codes).
2. Proportion of patients who received guideline-recommended medicines (ie, antiplatelet, statin, and angiotensin-converting enzyme inhibitors or angiotensin II receptor blocker), collected via linkage of the practice cohort with individual-level federal government administrative data for pharmaceutical prescriptions in Australia (via the Pharmaceutical Benefits Scheme) at 24 months.
3. Proportion of patients with a prepared General Practice Management Plan (MBS Item 721), prepared Team Care Arrangement (MBS Item 723), or an associated review (MBS Item 732) collected via linkage of the practice data (within the preceding 5 months) with individual-level federal government administrative data at 24 months. These plans are funded by the Australian Medicare system to support care planning and review in primary care.
4. Proportion of patients not achieving national targets for CVD risk factors (LDL ≥2 mmol/L, systolic BP >130 mm Hg, diastolic BP >80 mm Hg, and current smoker), collected via primary care clinical data extraction at 24 months.
5. Primary and secondary outcomes at 12 months from baseline.

Engagement with the intervention was summarized through an end-of-intervention user survey combined with submission of PDSA cycles and attendance at learning workshops.^20^ The user survey included Likert questions about usefulness and satisfaction, as well as free-text questions exploring barriers and enablers to engagement. Practice team members, including general practitioners, practice managers, or nurses from intervention practices who were actively involved in implementing data-driven improvements within their practices, were invited to complete the survey. De-identified survey responses were entered into an online database and analyzed using descriptive statistics. Free-text responses were coded using thematic analysis.

### Statistical Analysis

The target number of participating practices was 50. The target individual sample size was 6050 (3025 per group), with an average cluster size of 121 patients per practice. Using a 5% 2-sided test, this was estimated to provide 80% power to detect a relative risk of 0.75. This calculation assumed a control group readmission rate of 35% based on an Australian cohort study reporting an atherothrombotic disease readmission rate of 35% at 2 years for patients with CHD.^21^ An intraclass correlation coefficient of 0.05 was assumed, based on data from 2 cross-sectional studies in Australian primary care.^22^

Analysis was conducted at the individual patient level following the intention-to-treat principle with data analyzed according to the randomization group. Baseline patient characteristics, cardiovascular risk, and treatment usage were summarized using frequency and percentage for categorical variables and mean and SD for continuous variables. The primary analysis was conducted using the log-binomial regression within the framework of generalized estimating equations with an exchangeable correlation to account for the clustering of patients within general practices. The effect of the intervention is presented as a relative risk and 95% CI. A planned sensitivity analysis of time to unplanned CVD admission was performed using a Fine-Gray competing risks model with a robust sandwich covariance matrix estimate to account for the competing risk of death and to provide valid standard errors that are robust to possible misspecification of the assumed correlation structure, including potential clustering within general practices. These results are reported as subdistribution hazard ratio and 95% CI. The cumulative incidence curve has been plotted, which accounts for competing risks and shows the probability of experiencing the event over time. Two additional sensitivity analyses were also performed. The first was on the patients who had returned to the practice after baseline. The second sensitivity analysis adjusted for practice-level Index of Relative Socio-economic Advantage and Disadvantage in tertiles (low: 1–4 deciles; middle: 5–7 deciles; high: 8–10 deciles)^23^ and number of general practitioners, using a multilevel log-binomial regression model. An exploratory subgroup analysis was also undertaken to examine the effects of sex on the primary outcome. A post hoc minimum detectable difference was estimated using the observed event rates and design parameters to assist interpretation of the results of the primary outcome. Secondary outcomes were analyzed using the log-binomial regression within the framework of generalized estimating equations with an exchangeable correlation, reported as relative risk and 95% CI. All analyses were performed using a 2-sided significance level of 0.05 in SAS, version 9.4 (SAS Institute, Inc, Cary, NC).

## Results

**Figure 1.**
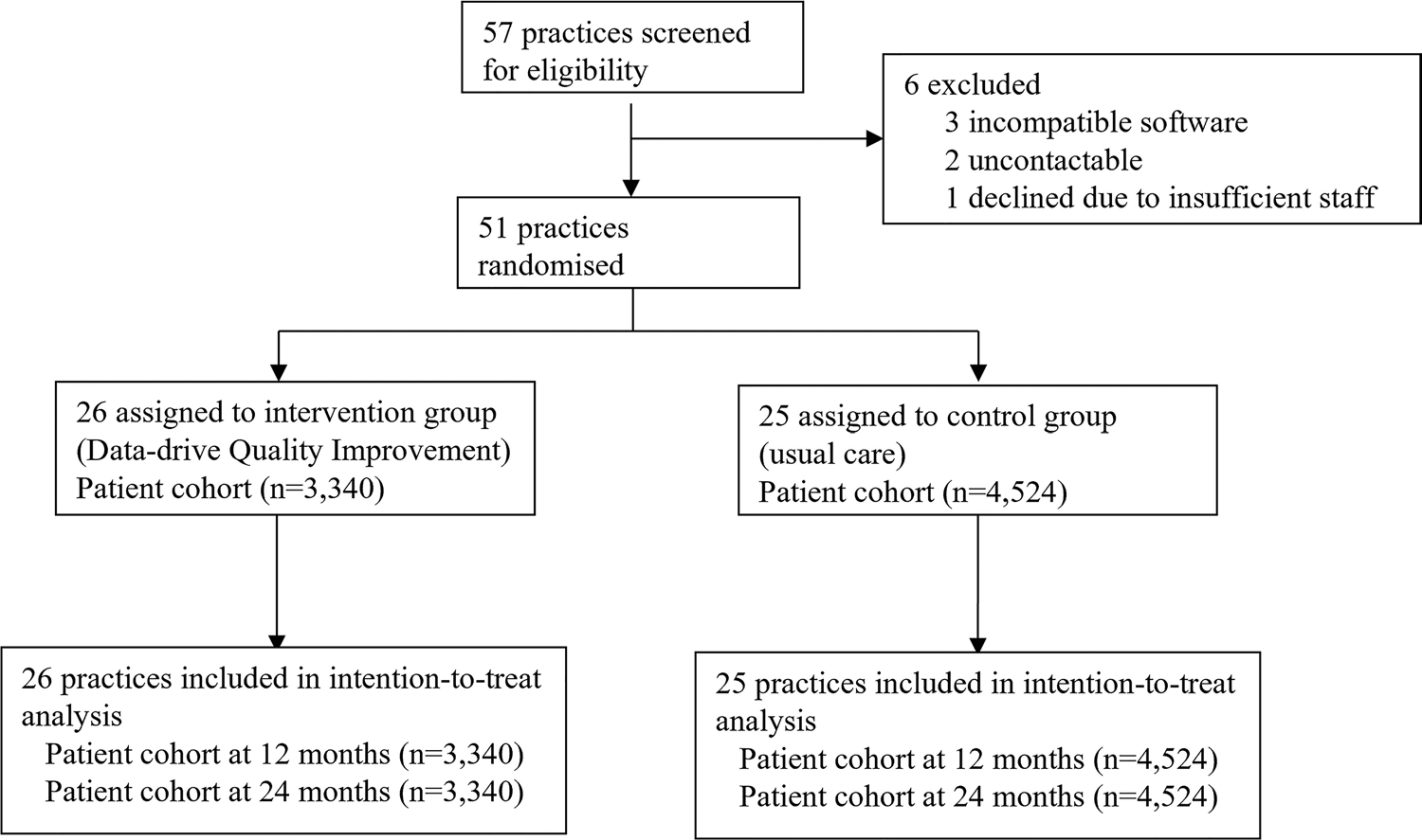
**Study flowchart for QUEL trial (Quality Improvement in Primary Care to Prevent Hospitalizations and Improve Effectiveness and Efficiency of Care for People Living with Coronary Heart Disease).** Flow of practices and the patient cohort through the study, including screening, eligibility, exclusions with reasons, enrollment, allocation to study groups, follow-up, and inclusion in the final analysis. Numbers of practices and patients at each stage are shown.

A total of 51 primary care practices participated in the QUEL trial, with baseline data collected on November 10, 2019, and final extraction on December 10, 2021. This resulted in an individual-level patient cohort of 7864; 4524 from 25 control practices and 3340 from 26 intervention practices (Figure 1), with a median number of eligible patients per practice of 187. All practices provided clinical data for linkage, so there was no loss to follow-up at 12 or 24 months for the primary outcome. Primary care clinical records (LDL, systolic BP, smoking status, body mass index) could not be collected at 12- and 24-month follow-up for all patients due to a change in the primary care electronic medical record software since baseline. However, all data were obtained for the primary outcome, MACE, medication prescriptions (Pharmaceutical Benefits Scheme), and General Practice Management Plan (MBS).

The practices in the control and intervention groups were well balanced for practice characteristics, socioeconomic status according to postcode, and median (interquartile interval) number of patients per practice (187 [129–470] versus 187 [110–318]), and number of general practitioners working at each practice (4 [2–9] versus 7 [3–10]) for the control and intervention groups, respectively (Table 2). In terms of the patient cohort, the groups were well balanced at baseline in terms of practice characteristics, patient demographics, and clinical measures but there were more patients with CHD attending control practices (Table 3).

**Table 2.**
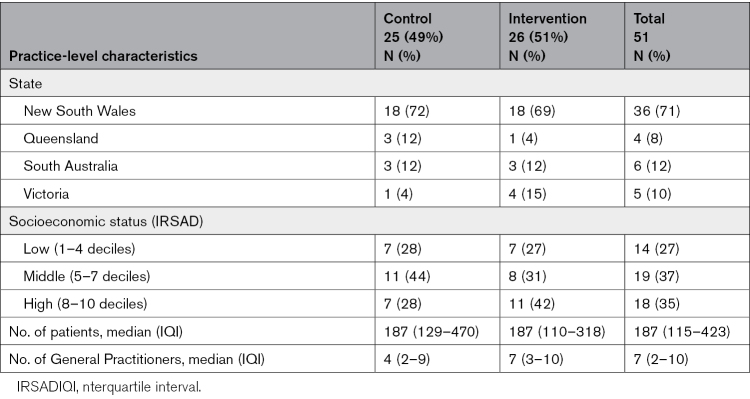
Practice-Level Characteristics

**Table 3.**
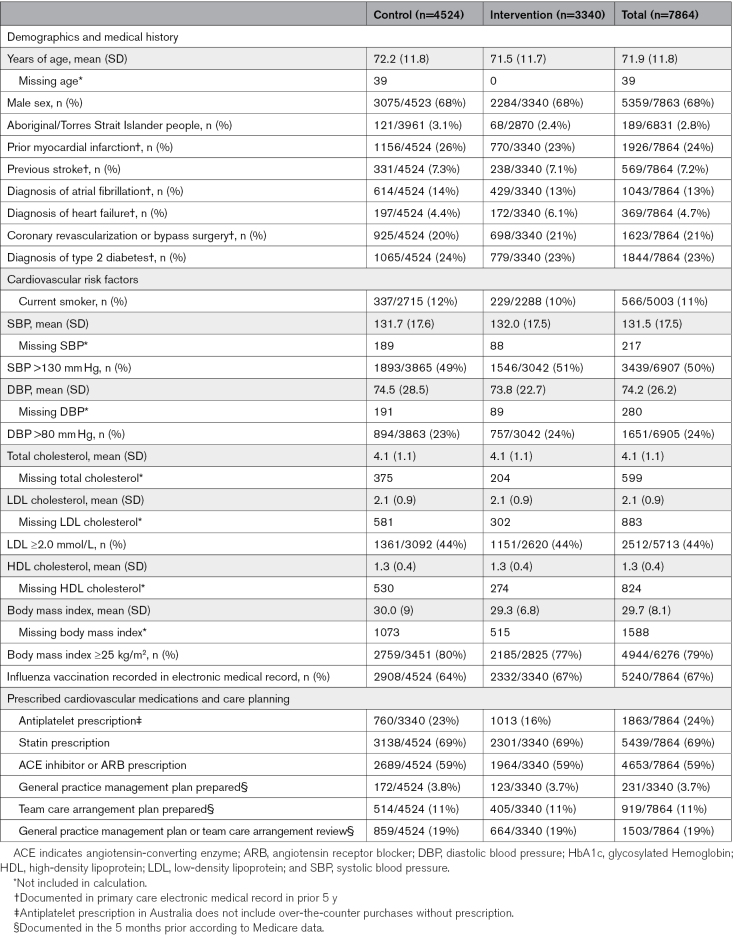
Baseline Demographics and Characteristics of Included Patients

For the primary outcome, the rate of unplanned CVD hospitalizations at 24 months was not significantly different between the control (11.5%) and intervention (10.6%) groups (relative risk, 0.91 [95% CI, 0.75–1.10]; Table 4). There were a total of 432 deaths, with no statistically significant difference between the control (270/4524; 6.0%) and intervention groups (162/3340; 4.9%) at 24 months. Similarly, there were no significant differences in secondary outcomes or influenza vaccination rates between the intervention and control groups (Table 4).

**Table 4.**
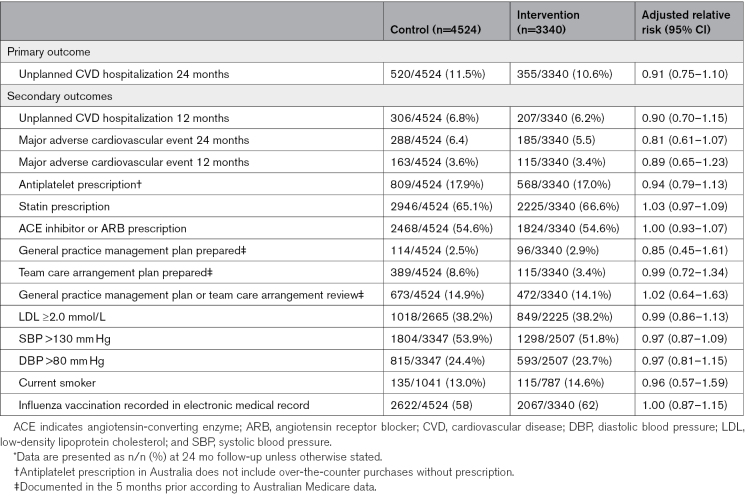
Analysis of Primary and Secondary Outcomes for Included Patients*

**Figure 2.**
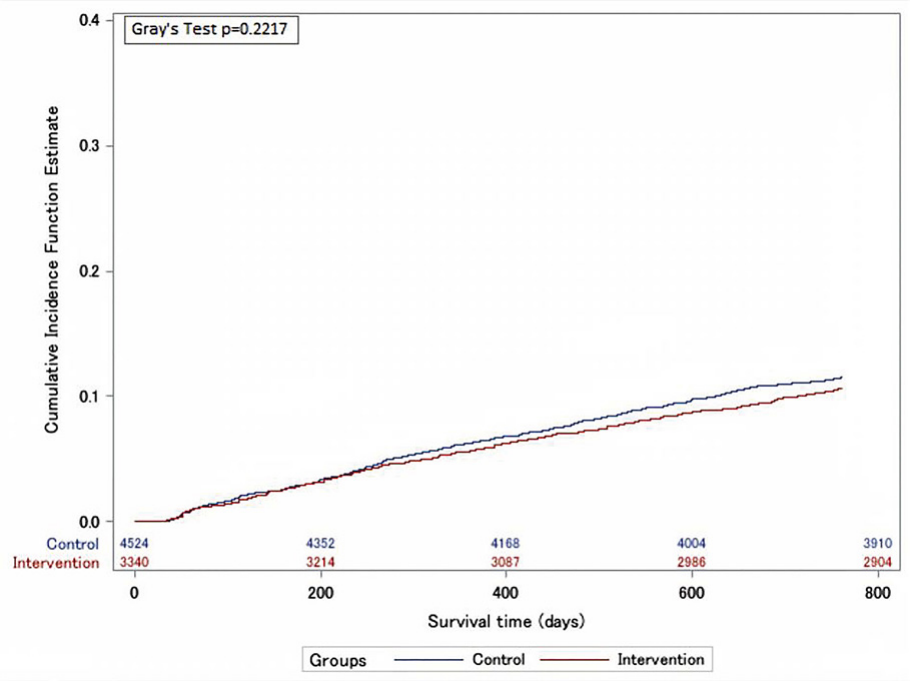
**Cumulative incidence curve of unplanned 2-year cardiovascular disease admission by control and intervention.** Cumulative incidence curves, accounting for the competing risk of death, were plotted to illustrate the probability of an unplanned hospital admission for cardiovascular disease over time for the control and intervention groups.

The planned sensitivity analysis to assess the effect of the intervention compared with the control on time to unplanned cardiovascular hospitalization, accounting for the competing risk of death, revealed that the difference remained nonsignificant (subdistribution hazard ratio, 0.92 [95% CI, 0.73–1.16]). A cumulative incidence curve is presented in Figure 2. The second sensitivity analysis on the 6601 (84%) patients who had revisited the practice after baseline showed similar results to the original finding (relative risk, 0.94 [95% CI, 0.76–1.15]). The third sensitivity analysis, which adjusted for practice-level factors showed no difference between the 2 groups (relative risk, 0.93 [95% CI, 0.76–1.13]). Subgroup analysis also found no difference in the effect of the intervention on the primary outcome compared with control between sexes (Table S2).

Using these findings, the minimum detectable difference was estimated. Using the event rate of 11.5% in the control group and the trial design parameters (25 control clusters, 26 intervention clusters, mean cluster size=187, ntraclass correlation coefficient =0.015, α=0.05, 80% power), the minimum detectable absolute difference was ≈3.6 percentage points. The observed 0.9 percentage-point difference was smaller than this threshold; therefore, there was limited statistical power to detect modest effects.

Of the practices allocated to the intervention, 85% (22/26) attended 3 or more of the learning workshops, and 69% (18/26) submitted at least 1 PDSA cycle. When asked about satisfaction with the learning workshops, 94% of participating practices rated Learning Workshop One (in-person) as highly satisfactory, and 76% rated Learning Workshop Six (virtual) as highly satisfactory. Further, 90% of practices reported that they were well informed about the objectives of the workshops, and 85% reported that they felt able to use what they learned in the workshops.

## Discussion

QUEL was an investigator-initiated cluster randomized clinical trial that tested the effectiveness of data-driven quality improvement to strengthen secondary prevention of CHD in primary care. It is arguably one of the largest and most robust studies evaluating the effectiveness of this type of intervention in primary care or an acute hospital setting. No statistically significant improvement was found in unplanned CVD hospitalizations, MACE, or intervention KPIs related to CVD risk factors or medication prescriptions at 12 and 24 months. The data-driven and collaborative approach aimed to promote shared learning and quality improvement among participating practices, and the majority of intervention practices participated in workshops and submitted PDSAs.

The QUEL intervention included all the standard aspects of a Breakthrough Series collaborative, with a similar intervention duration, number of sites, and length of follow-up to other studies evaluating similar interventions.^10^ Previous studies have reported positive process and qualitative findings, but the majority had weak designs and surrogate end points.^11^ Three relevant cluster randomized controlled trials have been conducted in primary care; they aimed to improve asthma care,^12^ colorectal screening,^24^ and diabetes care.^25^ The asthma care and colorectal screening trials both found no significant effect of the intervention on their primary (asthma action plans and colorectal screening rates, respectively)^12,24^ or secondary outcomes (including hospitalizations for the asthma trial).^12^ The diabetes trial found a significant within-group improvement, but analyses were not adjusted and did not directly compare between study groups.^25^ Another relevant, well-designed cluster randomized controlled trial evaluated collaborative quality improvement for heart failure care in a hospital setting and found no significant effect on heart failure processes of care or workforce.^13^ The QUEL trial included a large patient cohort and robust end points such as hospitalizations and MACE, and taken together, it is argued that the QUEL trial adds to the body of evidence suggesting data-driven and collaborative quality improvement may not be effective in improving clinical outcomes for people with complex health conditions in primary care.

There are various explanations for the findings of the QUEL study. First, the data-driven quality improvement strategy is not effective in reducing hospital admissions, cardiovascular events, and cardiovascular risk factors. The QUEL study was large in size and robust in design, and the results align with other similar studies and hence support a lack of evidence for this intervention. The intervention group did have a lower rate of CVD hospitalization at both 12 and 24 months, so it is possible that the difference did not reach significance due to the lower than expected hospitalization rate. It is also possible that not all intervention practices were as engaged as they may have needed to be, as evidenced by the low rate of PDSA submission and also because the intervention period fell within the COVID-19 global pandemic. Qualitative details about practice engagement are reported elsewhere and highlight varied engagement with learning workshops and monthly feedback reports, which were considered the most useful features of the intervention.^26^ Importantly, the QUEL study was directed at CHD, and despite expectations of this being a suitable area of health care for data-driven quality improvement, it is possible that the intervention may be better suited to other health conditions. Further robustly designed research could elucidate such evidence.

The QUEL trial provides insights for future decentralized clinical trials linking primary care data with other administrative health data. The Food and Drug Administration describe defined decentralized clinical trials as those where some or all trial-related activities occur at locations other than traditional clinical trial sites (eg, the participant’s home, mobile research units, or local health care facilities).^27^ This design is relevant to QUEL given the remote data collection and data-driven nature of the intervention, with virtual and telephone support to practices. QUEL offers insight into some of the strengths and weaknesses of such an approach. Strengths include the streamlined recruitment of a large cohort without individualized in-person visits (improved efficiency and reduced resources) and the minimal loss to follow-up.^28,29^ Further, QUEL demonstrated how Australian primary care data can be linked with jurisdictional and national data within the context of a clinical trial. This issue is particularly interesting in Australia, where hospitalization data are collected by state/territory governments yet the national government collects deaths and medication prescription data. Efforts to overcome challenges with sharing and linking individual-level patient data, as was the case for QUEL, could improve the efficiency of large-scale trials.

There are limitations associated with the QUEL trial. The collection of data via linkage is efficient and pragmatic but also means the quality of reported outcomes is dependent on the quality of data entered at the point of care. This could be an issue for the primary care data if practice-level data collection is not up to date. Administrative data for hospitalizations in Australia are collected at the state level, and hence if a person regularly attended a practice in an included state but was admitted to a hospital in a nonparticipating state, their admission may not have been captured. The likelihood of this occurring, however, is extremely low. It is possible that the COVID-19 pandemic negatively impacted the ability of intervention practices to prioritize data-driven quality improvement for secondary prevention of CHD. The pandemic may also have impacted hospitalization rates and study power given the observed rate of the primary outcome being much lower than anticipated.

## Conclusions

A 12-month data-driven quality improvement program in primary care did not result in improvement in unplanned hospitalizations, MACE, or medication prescriptions for people with CHD at 24 months. The trial was arguably one of the largest and most robust studies evaluating the effectiveness of this type of intervention in primary care or an acute hospital setting and does add to the growing body of studies showing a lack of evidence. The nature of the study design enabled the trial to have a large sample size with robust outcomes in an efficient way and provides insight for future trials. The use of a collaborative approach to improving care for people with CHD and using routinely collected primary care data offers opportunity, but evidence remains elusive.

## Article Information

### Author Contributions

Drs Redfern, Chow, Briffa, Gallagher, Reid, Hare, Zwar, Woodward, Jan, Atkins, Laba, Halcomb, L. Billot, Drs Usherwood, and Hyun developed the trial design and scientific oversight. Drs Redfern, Hafiz, Knight, Hespe, Atkins, Laba, and Usherwood led finalization of key performance indicators. Drs Redfern, Hafiz, Knight, Hespe, and Manandi supported and contributed to learning workshops and oversight of plan-do-study-act (PDSA) cycles. Dr Hyun completed the analysis. All authors have reviewed, contributed to, and approved the article.

### Disclosures

None.

### Supplemental Material

Tables S1–S2

## Supplementary Material


