# Supplementary material for: Effectiveness of Data-Driven Quality Improvement on Hospitalizations and Health Outcomes for People With Coronary Heart Disease in Primary Care (QUEL): A Cluster Randomized Controlled Trial With 24-Month Follow-Up
Source: Circ Popul Health Outcomes. 2026 Apr 15;19(5):e012904. doi: 10.1161/CIRCOUTCOMES.125.012904 (PMC13275088; doi:10.1161/CIRCOUTCOMES.125.012904)
Supplement: Supplementary file 1 [file hcq-19-e012904-s001.pdf]

SUPPLEMENTAL MATERIAL

Table S1. Diagnosis and medication codes used for outcomes					
Outcomes	Datasets	ICD-10-AM codes	Underlying cause of death (ICD-10)	ATC codes	MBS Item numbers
Cardiovascular disease	NSW, QLD, SA and VIC admitted patient data*	I48, I20, I21, I22, I23, I24, I25, I50, I42, I43, I11.0, I130, I13.2, I05, I06, I07, I08, I34, I35, I36, I37, I38, I39, I09.1, I09.8, I70, I71, I72, I73, I74, I77, I78, I79, Q22, Q23, Q24.87, E09.5, E10.51, E10.52, E11.51, E11.52, E13.51, E13.52, E14.51, E14.52, I60, I61, I62, I63, I64, G45, G46			
Major adverse cardiovascular events	NSW, QLD, SA and VIC admitted patient data* and National Death Index	I20, I21, I22, I23, I24, I25, I60, I61, I62, I63, I64, G45, G46	I00-I99		
Antiplatelets	Pharmaceutical Benefits Scheme			B01AC01-B01AC11, B01AC13, B01AC15-B01AC19, B01AC21-B01AC28, B01AC30, B01AC56	
Statins	Pharmaceutical Benefits Scheme			C10AA01-C10AA08, C10BA01-C10BA12, C10BX01-C10BX21	
Angiotensin-converting enzyme inhibitors and angiotensin receptor blockers	Pharmaceutical Benefits Scheme			C09AA01-C09AA16, C09BA01-C09BA09, C09BA12-C09BA13, C09BA15, C09BB02-C09BB07, C09BB10, C09BB12-C09BB13, C09BX01-C09BX07, C09CA01-C09CA10, C09DA01-C09DA04, C09DA06-C09DA10, C09DB01-C09DB02, C09DB04-C09DB09, C09DX01-C09DX08	

GP management plan	Medicare Benefits Schedule				721
Team care arrangement plan	Medicare Benefits Schedule				723
GP management plan or team care arrangement review	Medicare Benefits Schedule				732

NSW, New South Wales; QLD, Queensland; SA, South Australia; VIC, Victoria; GP, general practice; MBS, Medicare Benefits Schedule.
*Admitted Patient Data Collection, Queensland Hospital Admitted Patient Data Collection, South Australian Public Hospital Separations, Victorian Admitted Episode Database

Table S2. Subgroup analysis of unplanned 2-year CVD hospitalisation by sex						
**Outcome**	**Subgroup**	**Intervention N (%)**	**Control N (%)**	**RR (95% CI)***	**P-value**	**Interaction p-value***
Unplanned 2-year CVD hospitalisations	Female	111/1056 (11%)	179/1448 (12%)	0.84 (0.66, 1.06)	0.1365	0.3364
	Male	244/2284 (11%)	341/3075 (11%)	0.94 (0.76, 1.17)	0.5851	

RR, relative risk; CI, Confidence interval; CVD, cardiovascular disease.

*Accounted for the clustering effect of practices
